# Supramolecular complexation of phenylephrine by cucurbit[7]uril in aqueous solution

**DOI:** 10.1039/d4ra01910e

**Published:** 2024-04-23

**Authors:** Khaleel I. Assaf, Ayah N. Faraj, Eyad S. M. Abu-Nameh, Mohammad A. Alnajjar

**Affiliations:** a Department of Chemistry, Al-Balqa Applied University Al-Salt 19117 Jordan khaleel.assaf@bau.edu.jo; b Department of Biology and Chemistry, Center for Cellular Nanoanalytics, Universität Osnabrück 49069 Osnabrück Germany

## Abstract

Cucurbiturils (CB*n*) are known to establish stable host–guest complexes with a variety of drug molecules. Herein, the supramolecular complexation between cucurbit[7]uril (CB7) and phenylephrine hydrochloride is reported in aqueous solution. Phenylephrine forms inclusion complex with CB7 with high binding affinity (*K*_affinity_ = 4.0 × 10^6^ M^−1^), which allows for the development of a fluorescence-based sensing assay applying the dye displacement strategy. The structure of the host–guest inclusion complex is investigated by ^1^H NMR spectroscopy, in which complexation-induced chemical shifts indicate the immersion of the aromatic ring inside the hydrophobic cavity of CB7. Density functional theory (DFT) calculations support the ^1^H NMR results, and reveal that the complex is stabilized through intermolecular interactions between the polar groups on the phenylephrine and the carbonyl rims of CB7, as well as the hydrophobic effect. Moreover, preferential binding of phenylephrine in its protonated over the neutral form results in a complexation-induced p*K*_a_ shift.

## Abstract 2

Stable host–guest complexation between phenylephrine and cucurbit[7]uril macrocycle in water is reported.

## Graphical abstract

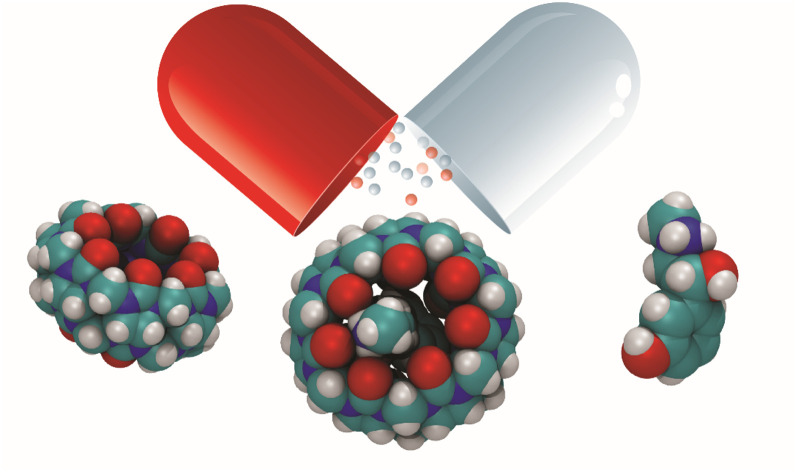

## Introduction

Cucurbit[*n*]urils, CB*n*, are a family of interesting macrocyclic host molecules that are water-soluble.^1,2^ CB*n* and other homologues are synthesized by the condensation of glycoluril and formaldehyde in strongly acidic media,^3,4^ where the reaction composition representing the molecules was first identified by crystallography.^5^ CB*n* are composed of *n* (5–8, 10 and 14) repeating glycoluril units linked *via* two methylene bridges on each side, which results in a barrel-shaped molecules with two identical carbonyl rims and lipophilic cavity with low polarity and polarizability.^1^ CB*n* have found great interest due to their ability to encapsulate a wide spectrum of guest molecules within the hydrophobic cavity with high association constants.^1,2^ The host–guest complexation of CB*n* is driven by the hydrophobic effect and non-covalent interactions.^6^ The relocation from water into the hydrophobic cavity of a macrocycle changes the physical and chemical properties of the encapsulated guests, such as solubility and stability.^7–10^ Therefore, CB*n* and their derivatives are suitable for several lines of applications including pharmaceutical, agriculture and chemical industries.^11–15^ Several examples on the complexation of drug molecules with different CB*n* hosts in aqueous solution are documented in the literature.^16^ For example, CB7 forms host–guest complexes with a variety of bioactive analytes such as amino acids, steroids, and drug molecules.^7,17–19^

**Fig. 1:**
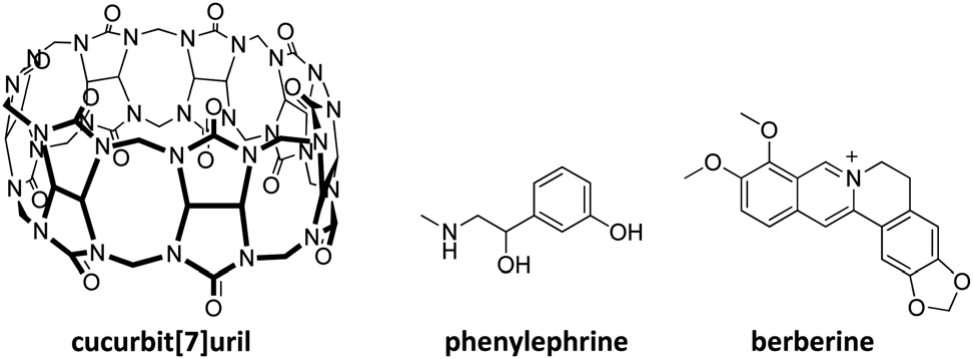
Chemical structure of cucurbit[7]uril (CB7), phenylephrine, and berberine dye.

In this work, we study the molecular recognition of phenylephrine by CB7 through host–guest complexation (Fig. 1). Indeed, CB7 was selected due to its moderate cavity size (242 Å) that fits small organic molecules, as well as its relatively high water solubility.^20^ Phenylephrine is used effectively to treat congestion, vasomotor rhinitis, hemorrhoids and hypotension, and commonly prescribed an alternative to pseudoephedrine.^21,22^ The supramolecular complexation is investigated by using different spectroscopic techniques and the structure of the host–guest complex is elucidated by nuclear magnetic resonance (NMR) spectroscopy and verified by quantum-chemical calculations.

## Results and discussion

**Fig. 2:**
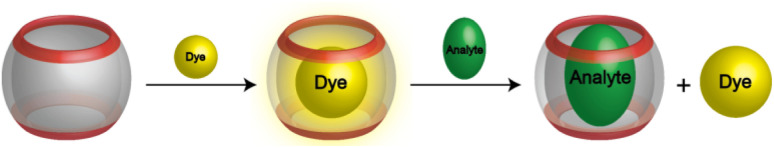
Schematic representation of the fluorescent-dye displacement assay principle for analyte binding using CB*n*/dye reporter pair.

CB*n* have been widely used to complex drug molecules and other biologically relevant analytes, due to their low toxicity.^23–25^ Phenylephrine is structurally similar to catecholamines, a class of important pharmacological molecules that act as neurotransmitters and hormones.^26^ The supramolecular complexation of catecholamines, such as dopamine, norepinephrine, and epinephrine with different macrocyclic hosts has been subject for several studies.^27–29^ The encapsulation of a guest molecule (*e.g.*, a dye) inside the hydrophobic cavity alerts its photophysical properties, including the absorption and fluorescence emission.^30–32^ This has extensively been applied to probe the host–guest complexation and allowed for the determination of the binding affinity of optically transparent guest molecules.^7,33–35^ Phenylephrine showed an absorption maximum in UV-range (*λ*_max_ = 275 nm, in acidic solution and *λ*_max_ = 290 nm, in basic solution). The addition of CB7 to a phenylephrine solution resulted in a hyperchromic shift, which in part a consequence of the CB7 absorption in the same region. Therefore, indicator displacement assay was chosen to determine the binding affinity of phenylephrine to CB7.^33,36^ Berberine chloride (Fig. 1) was selected as a sensitive dye, which is known to form stable inclusion complex with CB7.^37^ In aqueous solution, berberine showed a weak fluorescence emission (*λ*_em_ = 495 nm), which is significantly enhanced upon complexation with CB7, due to the relocation into the hydrophobic cavity and the rigid confinement.^37^ The binding constant of berberine was determined as 1.6 × 10^7^ M^−1^, in a good agreement with reported values.^37–39^ The preformed CB7·berberine complex can potentially be used as a reporter pair for the sensing of optically transparent guest molecules.^34^ The addition of a competitive binder (*e.g.*, phenylephrine) results in the displacement of the dye (*i.e.*, berberine) and the restoration of the original fluorescence emission. Fig. 2 illustrates the principle of fluorescence-based dye displacement.

**Fig. 3:**
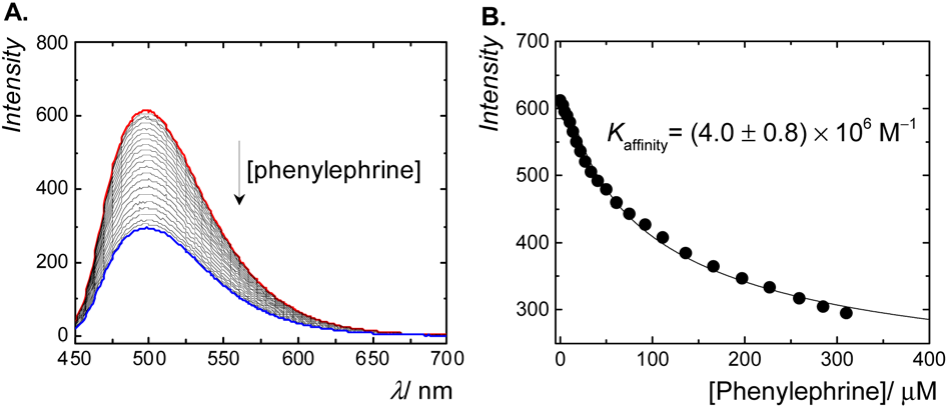
(A) Fluorescence emission spectra of CB7·berberine complex (*λ*_ex_ = 345 nm) at various concentrations of phenylephrine as a competitive guest in aqueous solution, at pH = 3.0. (B) The intensity changes (*λ*_em_ = 495 nm) as a function of phenylephrine concentration; the binding constant value was extracted from the fitted data as 4.0 × 10^6^ M^−1^.

The fluorescence displacement titration of phenylephrine is shown in Fig. 3A. The fluorescence intensity of complexed berberine decreased upon the addition of phenylephrine. This indicates the displacement of berberine by phenylephrine. The emission intensity was then monitored as a function of the phenylephrine concentration and fitted according to a 1 : 1 binding model, which resulted in a binding constant of 4.0 × 10^6^ M^−1^ (Fig. 3B). The binding affinity of phenylephrine to CB7 is found to be higher than that of dopamine^40^ (a structurally similar catecholamine), which might be attributed to the position of a hydroxyl group at the side chain of phenylephrine rather than at the phenyl ring in dopamine.

**Fig. 4:**
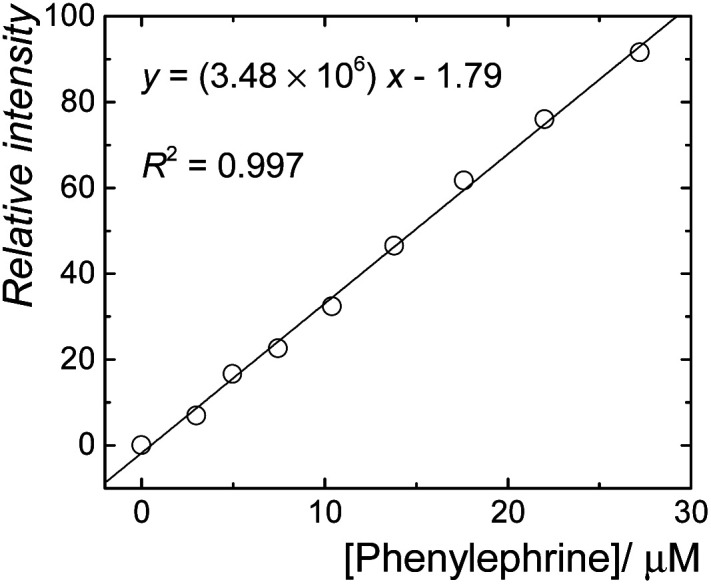
Linear fluorescence response for the detection of phenylephrine in aqueous solution using CB7·berberine reporter pair.

The sensitive fluorescence response of CB7·berberine to the phenylephrine could potentially be used for sensing purposes in aqueous solutions. A linear fluorescence response was observed in the concentration range of 0 to 30 μM of phenylephrine (Fig. 4, *R*^2^ = 0.997). Accordingly, the limits of detection (LOD) and quantification (LOQ) were estimated as 0.58 and 1.77 mg L^−1^. This represents a new fluorescence-based sensing method for phenylephrine.

**Fig. 5:**
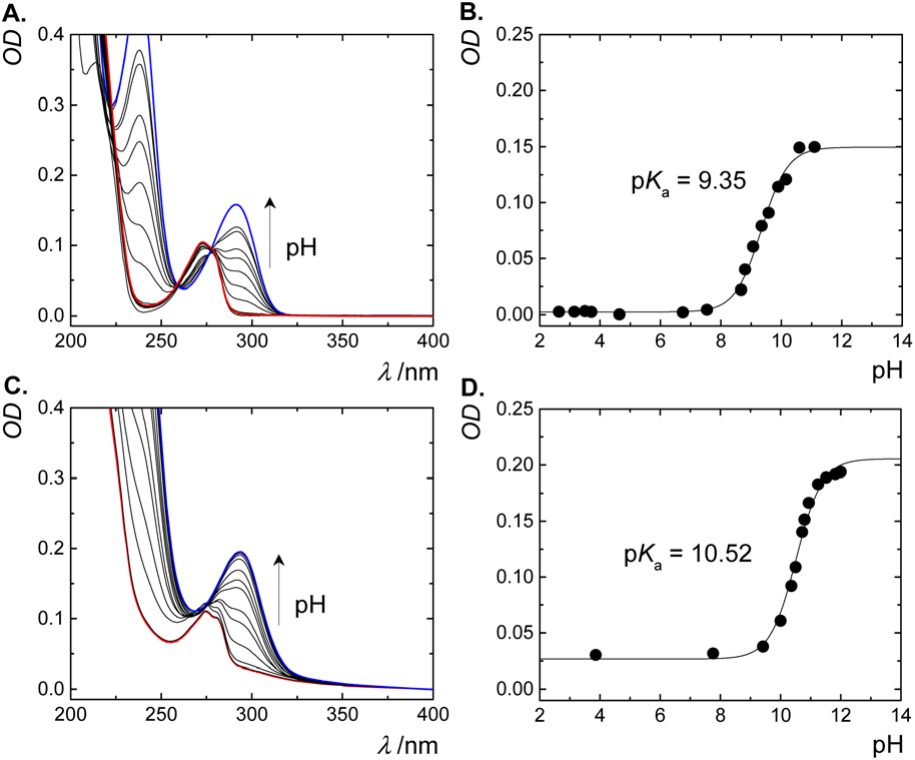
UV-absorption spectra of free phenylephrine and CB7·phenylephrine inclusion complex at different pH values (A and C, respectively). Fitted pH-dependent absorbance data at 295 nm (B and D).

CB*n* are known as strong binders for a variety of neutral and cationic guest molecules in aqueous solution.^2^ The preferential binding of the cationic form of guest molecules (*e.g.*, ammonium-based) over their neutral form, modulates their acid dissociation constants (p*K*_a_), leading to a complexation-induced p*K*_a_ shifts.^9,15,41,42^ The p*K*_a_ values of free and complexed phenylephrine were determined by monitoring the absorbance as a function of pH (Fig. 5). The UV-spectra of free phenylephrine showed an absorption band with *λ*_max_ of 275 nm at low pH (<7), which gradually shifts to a longer wavelength (bathochromic shift) as a function of increasing the pH (*λ*_max_ of 290 nm), with two isosbestic points at 259 and 278 nm. The p*K*_a_ of phenylephrine was determined as 9.35. Similarly, the p*K*_a_ of complexed phenylephrine was measured in the presence of 1.0 mM CB7 to ensure a high degree of complexation at elevated pH values. No significant change in the *λ*_max_ of phenylephrine was observed in the presence of CB7 in acidic solution, while a slight bathochromic shift (5 nm) was obtained in basic solution. CB7-complexed phenylephrine showed an increase in the p*K*_a_ value to 10.52 (Δp*K*_a_ of 1.17). This shift to a higher p*K*_a_ value indicated an increased basicity of phenylephrine in the presence of CB7 as consequence of the stabilization of the protonated form due to the ion–dipole interactions between the ammonium group in the phenylephrine and the carbonyl rim of CB7 (see below).

**Fig. 6:**
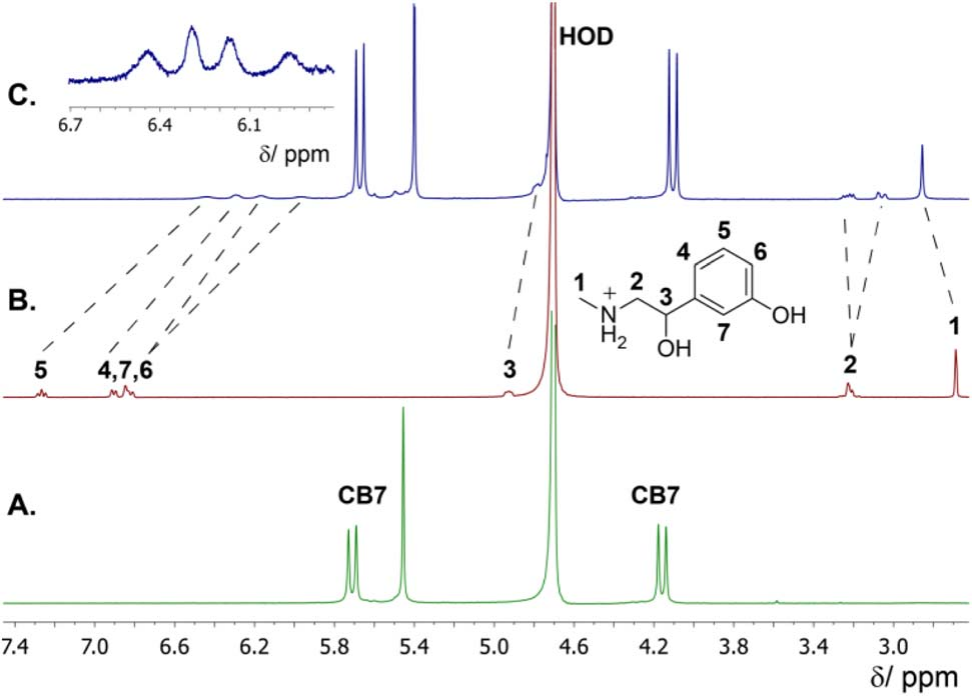
^1^H NMR spectra of (A) free CB7 (2.0 mM), (B) free phenylephrine (2.0 mM), (C) CB7·phenylephrine complex (1 : 1) in D_2_O.

The formation of host–guest complexes between phenylephrine and CB7, in acidic aqueous solution, was investigated using ^1^H NMR spectroscopy, which is a powerful technique used to extract structural information. The formation of inclusion complexes in aqueous solution is usually associated with complexation-induced chemical shifts.^43^ In detail, we rely on chemical shift values that indicate whether the guest molecule or part of it is located inside the hydrophobic cavity or closer to the portals of the macrocyclic host. This is usually obtained by measuring the difference in ^1^H NMR chemical shifts of the bound (*δ*_bound_) and free guest (*δ*_free_), which can be expressed as follows Δ*δ* = *δ*_bound_ − *δ*_free_. In the case of CB*n*, the host protons are less informative, due to the absence of protons inside the cavity. Changes in the chemical shift of the guest protons can give a clear evidence for complexation. When, Δ*δ* > 0, this means that the protons of the guest are located near to the carbonyl groups of CB7 and appear downfield shifted. When Δ*δ* < 0, the protons of the guest are positioned inside the lipophilic cavity of CB7 and appear upfield shifted. Fig. 6 displays the ^1^H NMR spectra of free phenylephrine, free CB7, and their complex, dissolved in D_2_O. The phenylephrine signals were assigned as shown in the chemical structure Fig. 6B. The formation of the inclusion complex between CB7 and phenylephrine affects the proton chemical shifts based on their location within the cavity of CB7. The relocation of the guest into the hydrophobic cavity of CB7 induced significant upfield shifts due to the shielding effect. In contrast, protons that are located at the carbonyl rims are usually downfield shifted compared to the uncomplexed ones. The aromatic protons 4–7 were upfield shifted upon complexation (Δ*δ* = −0.6 to −1 ppm), which indicated that the phenyl ring is positioned inside the cavity of CB7. Similarly, proton 3 experienced an upfield shift. In contrast, the methyl protons (1) were downfield shifted as expected from their location near the CB7 portal. The methylene protons (2) split upon complexation, which revealed different environments within the complex. These data revealed that the protonated ammonium group will be docked at the portal to enable ion–dipole interactions.

**Fig. 7:**
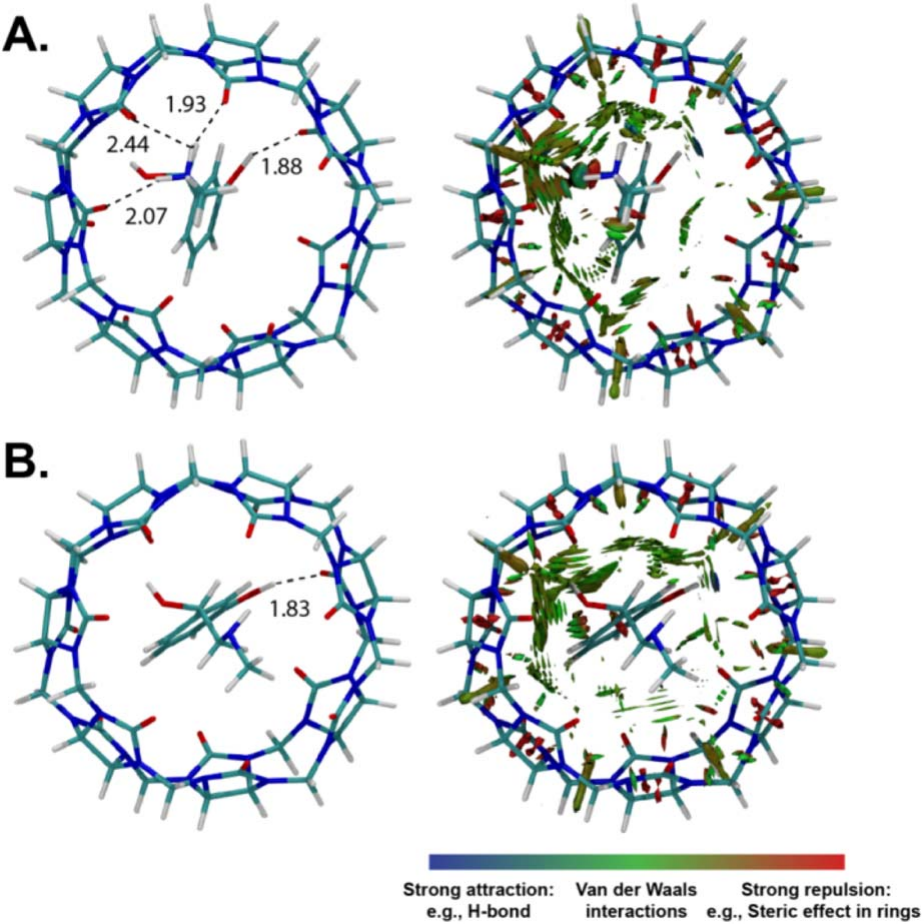
DFT-optimized structures of CB7·phenyephrine complexes (left) and gradient isosurface plots of evidencing non-covalent interactions (NCI, right). Hydrogen bonds are given in Å. (A) Protonated phenylephrine and (B) neutral phenylephrine.

Density functional theory (DFT) calculations were performed to get additional structural information on the host–guest complexation. The optimized structures for the protonated/neutral phenylephrine as well as the non-covalent interactions (NCI) analysis are shown in Fig. 7. NCI analysis provide the visual illustration of the non-covalent interactions (Fig. 7B), such as hydrogen bonds (depicted with blue color), van der Waals interactions (depicted with green color), and repulsive steric interactions (depicted with red color).^44^ The optimized structure of CB7·phenylephrine in the protonated form indicated the formation of inclusion complex, in which the phenyl ring is encapsulated inside the cavity. In addition, the complex is stabilized by ion–dipole interaction between the ammonium group and the carbonyl rim from the upper side, as well as hydrogen bonding between the hydroxyl group and the lower rim (Fig. 7A). This result is in agreement with the ^1^H NMR data. For the unprotonated form of phenylephrine, the inclusion complex is stabilized by hydrogen bonding between the amine/hydroxyl groups and the carbonyl rims (Fig. 7B). It can be seen from the NCI analysis that the protonated form of phenylephrine involved more attraction interactions compared to the neutral one. The calculated interaction energy revealed a stabilization of the protonated complex by 63 kcal mol^−1^ relative to the neutral one.

Phenylephrine hydrochloride extended stability at low and ready-to-use concentrations has been recognized problematic.^22,45,46^ Consequently, there is a need to improve stability phenylephrine hydrochloride. In this regard, supramolecular complexation provides a versatile solution for drug stability.^47^ The host–guest complexation between CB*n* and several drug molecules has shown positive impact on their stability.^16^ Therefore, the complexation of phenylephrine hydrochloride by CB7 may serve as a potential strategy to enhance its stability and bioavailability.

## Conclusions

In summary, we report on the host–guest complex between CB7 and phenylephrine in aqueous solution. CB7 forms inclusion complex with protonated phenylephrine with a high binding affinity, which is attributed to the combination of ion–dipole and hydrogen bonding interactions, as well as the hydrophobic effect. The supramolecular host–guest assembly resulted in a complexation-induced p*K*_a_ shift, revealing an increase in the basicity of phenylephrine. The recognition of phenylephrine by CB7 enables the development of fluorescence-based sensing assay applying the indicator displacement strategy.

## Experimental details

CB7 was synthesized as previously reported.^48^ Phenylephrine hydrochloride and berberine chloride were purchased as a solid powder from Sigma-Aldrich (Germany). Hydrochloric acid and sodium hydroxide were used to adjust the pH. UV-Visible absorption measurements were performed with a Varian Cary 4000 spectrophotometer and the fluorescence spectra were recorded on a Varian Cary Eclipse. All optical measurements were performed at ambient temperature, using a rectangular quartz cuvette with 1 cm optical path length. Proton nuclear magnetic resonance (^1^H NMR) spectra were measured by using a 400 MHz FTNMR NanoBay spectrometer (Bruker, Switzerland) in D_2_O.

Solutions were prepared in Milli-Q water and left to equilibrate before the measurements. The binding affinity of berberine dye to CB7 was measured by using fluorescence titration (pH = 3.0). The fluorescence emission (*λ*_ex_ = 345 nm and *λ*_em_ = 495 nm) of berberine (20 μM, kept fixed during the titration) was measured as a function of CB7 concentration and the binding constant (*K*_affinity_) was fitted according to the non-linear curve fit for a 1 : 1 binding stoichiometry.^49^ Indicator displacement experiment was performed to determine the binding constant of phenylephrine with CB7. In detail, phenylephrine was added to a solution of preformed CB7·berberine complex (using 35 μM of CB7 and 20 μM of berberine, both kept fixed during the titration), and the fluorescence emission (*λ*_ex_ = 345 nm and *λ*_em_ = 495 nm) was recorded upon each addition. The data were fitted according to the non-linear curve fit.^50^ For the p*K*_a_ determination, the absorption spectra of phenylephrine (50 μM) were recorded as a function of pH in the absence and presence of 1.0 mM CB7 (to ensure a high degree of complexation).^51^^1^H NMR spectra for free CB7 (2.0 mM) and phenylephrine (2.0 mM), as well as the CB7·phenylephrine complex were measured in D_2_O.

Quantum-chemical calculations were performed using Gaussian 16 ^52^ at the M06-2X/6-31+G** level of theory in the water applying the implicit solvation model based on density (smd) method.^53^ The non-covalent interactions (NCI) have been performed by using MULTIWFN v3.7.^44^

## Author contributions

K. I. A. conceptualization, methodology, supervision, writing – review and editing. A. N. F. data curation, formal analysis, visualization, E. S. M. A. supervision, writing – review and editing. M. A. A. data curation, formal analysis, visualization, writing – review and editing.

## Conflicts of interest

There are no conflicts to declare.

## Supplementary Material
